# A THEORETICAL FRAMEWORK FOR UTI PREVENTION AND MANAGEMENT IN COMMUNITY-DWELLING OLDER PERSONS WITH DEMENTIA

**DOI:** 10.1093/geroni/igac059.2776

**Published:** 2022-12-20

**Authors:** Kuan-Ching Wu, Oleg Zaslavsky

**Affiliations:** University of Washington, Seattle, Washington, United States; Unversity of Washington, Seattle, Washington, United States

## Abstract

UTI admission rates are 3.4 times higher among persons with dementia (PWD) than among those without dementia. The hospitalizations often result in adverse health complications such as the increased risk of delirium, cognitive decline, and higher mortality. To date, several UTI guidelines exist for specific populations, including women, children, people with catheters, and long-term care patients; however, there has been little focus on non-pharmacological prevention and management in PWD. The goal of this work is to propose a theoretical framework for UTI prevention and management in community-dwelling older PWD with the following three aims: 1) identify factors and outcomes of UTIs in community-dwelling older PWD; 2) generate a conceptual framework informed by Bronfenbrenner’s 1977 ecological model; 3) integrate preventive measures to the adapted framework. We conducted a scoping literature review utilizing the following keywords and concepts: risk factors, UTI management and prevention, community-dwelling older adults, and dementia. The UTIP framework was generated with four levels of risk factors (individual, interpersonal, community, and national) and four domains of affected outcomes (PWD, caregivers, PWD and their caregivers, and community/national). Additionally, seven themes of preventive measures emerged: physical activity and mobility, behavioral therapy, personal hygiene, phytotherapy, dietary, compound, and hormone. Finally, a table of care management opportunities was proposed according to dementia severity and caregivers’ involvement. In conclusion, the UTIP framework synthesizes exciting and is flexible to accommodate new evidence. As such, the framework is informative for programs, practices, and policies to lower the risks of preventable admission in PWD.

